# Gene targeting, genome editing: from Dolly to editors

**DOI:** 10.1007/s11248-016-9932-x

**Published:** 2016-02-03

**Authors:** Wenfang Tan, Chris Proudfoot, Simon G. Lillico, C. Bruce A. Whitelaw

**Affiliations:** The Roslin Institute and Royal (Dick) School of Veterinary Studies, University of Edinburgh, Easter Bush Campus, Midlothian, EH25 9RG UK

**Keywords:** Gene targeting, Genome editing, Livestock, TALENs, CRISPR/Cas9, SCNT or cloning, Cytoplasmic injection

## Abstract

One of the most powerful strategies to investigate biology we have as scientists, is the ability to transfer genetic material in a controlled and deliberate manner between organisms. When applied to livestock, applications worthy of commercial venture can be devised. Although initial methods used to generate transgenic livestock resulted in random transgene insertion, the development of SCNT technology enabled homologous recombination gene targeting strategies to be used in livestock. Much has been accomplished using this approach. However, now we have the ability to change a specific base in the genome without leaving any other DNA mark, with no need for a transgene. With the advent of the genome editors this is now possible and like other significant technological leaps, the result is an even greater diversity of possible applications. Indeed, in merely 5 years, these ‘molecular scissors’ have enabled the production of more than 300 differently edited pigs, cattle, sheep and goats. The advent of genome editors has brought genetic engineering of livestock to a position where industry, the public and politicians are all eager to see real use of genetically engineered livestock to address societal needs. Since the first transgenic livestock reported just over three decades ago the field of livestock biotechnology has come a long way—but the most exciting period is just starting.

## Introduction

Biology has many facets and our ability to utilize novel biological pathways increases every year. As scientists we strive to develop tools and strategies to help us tease apart biological process so we can better understand them. To achieve this understanding, biologists often turn to the powerful approach involving gene transfer enabling the consequence of alterations in gene activity to be studied in vivo. Since the first steps in the early 1970s involving the transformation of bacteria, the successful transfer of genes into first mammals and then plants quickly followed, with similar progress in fish and insects soon achieved. The first transgenic livestock announced in the mid-1980s (Hammer et al. 1985) followed the pioneering work of Palmiter and Brinster in mice (Brinster et al. 1981). Since then we have come a long way, with an explosion of activity in recent years.

The field of genetically engineered livestock has been driven by technological advances. This 30 year journey started slowly, with pronuclear injection (PNI) being the first tool in the kit (Hammer et al. 1985). Although conceptually simple—delivering DNA by injection through a fine glass needle into one of the pronuclei of a fertilised egg—this method is technically demanding and those who could successfully accomplish it were given great respect by the research community. PNI was king for the first decade of transgenic livestock research, with the commercial enterprises emerging on the back of this technique focused on producing human biomedical proteins in animal bioreactors (Jänne and Alhonen 1998; Kind and Schnieke 2008). But it has limitations. Efficiency of generating founder animals was low, and the injected DNA construct integrated randomly into the genome resulting in unpredictable transgene expression profiles. The field needed to progress.

Just over 10 years ago, oncoretroviruses were first employed to produce transgenic livestock (Chan et al. 1998; Cabot et al. 2001). While harnessing the innate ability of these replication defective viral vectors to transduce the livestock zygote dramatically increased the efficiency with which founder transgenic animals could be created, it quickly became obvious that there were issues with silencing of transgene expression in subsequent generations. The move from oncoretroviruses such as MoMLV to lentiviruses increased the transgenesis efficiencies further; Whitelaw et al. (2004) used an EIAV-based virus encoding eGFP to produce 40 founder piglets, 37 of which were transgenic and 35 of which expressed eGFP. Breeding from a subset of these for four generations revealed no loss of gene expression as assessed by visual GFP fluorescence and western blot for GFP protein (Whitelaw et al. unpublished data), in contrast to other studies with lentivirus transgenes where gene silencing was observed as assessed by loss of GFP fluorescence and DNA methylation (Hofmann et al. 2006). This impressive efficiency has allowed lentiviruses to be used to create a cohort of transgenic founder animals to model human disease (Kostic et al. 2013). However, viral vectors remain limited to a small cargo carrying capacity (around 8 Kb for lentiviruses) and the fact that they can only act as unidirectional delivery vehicles. Offering comparable efficiencies, transposon-based transgenesis (Carlson et al. 2011; Jakobsen et al. 2011) is less constrained with regard to vector design and the vagaries of transgene silencing (Ivics et al. 2014).

## Gene targeting by homologous recombination

Those working on transgenic livestock looked around to see what was being achieved in other mammalian species. In particular attention was drawn to what was technically possible in rodent research, where in addition to random transgene integration through pronuclear injection, the ability to perform gene targeting was possible. Gene targeting is made possible through homologous recombination (HR) which involves the exchange of nucleotides between two similar or identical DNA sequences (Capecchi 1989). In this way a gene can be targeted for disruption, termed knockout (KO), or used as a docking site for transgene insertion, termed knock in (KI)—this achievement attracted the first of two Nobel Prizes in this field.

HR in mammalian cells is an inefficient process, so relied on inclusion of a selectable marker in the construct to enable only the cells containing gene targeted events to survive. Subsequently more elegant strategies involving recombination-steps, driven for example by Cre recombinase (Nagy et al. 2009), enabled removal of the undesired marker gene. The latter has also enabled targeting of a transgene to a given genetic ‘harbour’, usually intended as a site permissive for expression of the transgene (Bronson et al. 1996; Wallace et al. 2000). The multiple steps involved and the low targeting efficiencies achieved meant that HR was not practical in zygotes; a cell-based system was required and the mouse research community had a very good one. Embryonic stem cells (ESCs), derived from the preimplantation embryo, have the ability to both self-renew and retain pluripotential characteristics (Torres-Padilla and Chambers 2014; Martello and Smith 2014). The first property facilitates the lengthy process of gene targeting while the latter allows the engineered cell to contribute to the germline after transfer into the early embryo (Capecchi 1989).

HR and ESCs transformed mouse-based research in the 1980s and until recently formed the mainstay for this research community, enabling the development of huge and important research resources. The race was on to achieve similar progress in livestock. But there was a hurdle. Livestock ESCs had not and still have not been isolated (Malaver-Ortega et al. 2012). For reasons that remain a mystery no robust livestock ESCs have ever been demonstrated, even though huge advances in our understanding of both rodent and human ESC biology (Torres-Padilla and Chambers 2014) and the requirements for their maintenance in culture has been achieved (Buehr et al. 2008). An alternative method of producing ESC-like cells through a process involving regression of differentiated cells was developed in 2006 (Takahashi and Yamanaka 2006). These induced pluripotent stem cells (iPSC) can self-renew and like ESCs can differentiate into many different cells types including the germline but are not derived from the early embryo. Again this development was pioneered in mice—winning the second Nobel Prize in the field—and was quickly transferred into human biology. However, while there have been some limited successes in livestock, iPSCs remain surprisingly difficult to isolate and even harder to maintain (Telugu et al. 2011; Ezashi et al. 2012; Nowak-Imialek and Niemann 2012).

## Cloning first enabled gene targeting in livestock

Since robust pluripotent livestock cells have to-date proven beyond reach, the livestock research community had to come up with an alternative—which they did in the mid-1990s. That alternative manifested in the birth of Dolly the sheep (Wilmut et al. 1997). Next year will be the 20th anniversary of Dolly and in these intervening years considerable use of somatic cell nuclear transfer (SCNT), more commonly referred to as cloning, has been the method of choice for many teams engaged with producing transgenic livestock (Kues and Niemann 2011; Prather 2013; Wolf et al. 2014). SCNT utilises primary cells grown in culture. During this in vitro phase manipulation of the donor genome utilising methodologies based on HR can be applied, with the selected transgenic cell then used to reconstitute an enucleated oocyte. The resulting transgenic animals are clones, derived from genetically identical parental cells. SCNT has enabled transgenic livestock research to develop since the late 1990s. Nevertheless, even with technical advances to simplify the technique such as handmade cloning (Peura and Vajta 2003), SCNT remains technically difficult and only a few labs around the world have truly mastered it.

By the turn of the millennium we were able to do gene addition, gene KO and gene KI—the latter two only by SCNT—in livestock. Given the cost involved in these studies, the need for SCNT and the complexity of HR driven gene targeting, transgenic livestock research has focussed primarily on biomedical applications. Here notable progress has been made in the extent of resources now available for xenotransplantation applications (Klymiuk et al. 2010; Satyananda et al. 2013; Cooper et al. 2014) and the commercial success of animal bioreactor derived products (Bösze et al. 2008). Agricultural applications, however, have lagged behind primarily due to concerns over public acceptance of livestock containing transgenes in the food chain.

## The revolution that is genome editors

1996 was a watershed year for the generation of engineered animals; not only did it include the birth of Dolly but also the generation of the first programmable nuclease (Kim et al. 1996). The former was trumpeted loudly by the media with reverberations still echoing around this field. It was over a decade for the latter to emerge as the technical revolution it is. Nearly 20 years on we now have a variety of tools and techniques at our disposal for the generation of engineered livestock species. Until recently we have only been able to dream of the ability to change a specific base in the genome without leaving any other DNA footprint; or the ability to induce precise insertions or deletions easily and efficiently in the germline of livestock. With the advent of the genome editors this is now possible.

Designer nucleases are used to generate a double strand break (DSB) at a desired genomic locus and can be divided into two broad categories; synthetic and natural. The first category includes the original zinc finger nuclease (ZFN; Kim et al. 1996) and the newer transcription activator like effector nuclease (TALEN; Christian et al. 2010) both of which are modular proteins containing an adaptable DNA binding domain fused to the nuclease domain of FokI. In the case of ZFNs each individual zinc finger binds three DNA bases whereas each TAL repeat binds a single base. Both ZFNs and TALENs are employed as pairs which recognise opposing DNA strands and orientate such that their fused FokI monomers are brought together on the intervening sequence to form an active enzyme dimer that cleaves both strands. In a refinement of this system the FokI monomers have been mutated such that heterodimerisation is obligate for FokI cutting (Miller et al. 2007; Doyon et al. 2011).

The second category includes meganucleases (Smith et al. 2006) and the newest, and currently the most popular of the designer nucleases, the clustered regularly interspaced short palindromic repeat/CRISPR associated gene (CRISPR/Cas) system (Cong et al. 2013; Mali et al. 2013; Jinek et al. 2013). Uptake of meganucleases by the livestock research community has not been widespread, presumably due to the laborious protein re-design and optimisation that is required to repurpose these molecules to a novel DNA sequence (Smith et al. 2006), however, effort to develop this nuclease tool continues (Ménoret et al. 2013). In contrast, the CRISPR/Cas system, which was first described just over 2 years ago, has seen an unprecedented exponential increase in its use (Seruggia and Montoliu 2014). This relatively simple system is adapted from an innate immune mechanism common to many bacteria and archaea, the function of which is to protect against invading viruses. The most widely used system at present is based on the CRISPR/Cas9 of Streptococcus pyogenes and involves a short guide RNA (sgRNA) sequence complexed with Cas9 nuclease. Specificity is determined by hybridisation between the 20 ribonucleotides of the complexed Cas9/guide and the nascent DNA target sequence, further restricted to sites immediately proximal to a protospacer adjacent motif (PAM) sequence (Cong et al. 2013; Mali et al. 2013; Jinek et al. 2013).

**Fig. 1:**
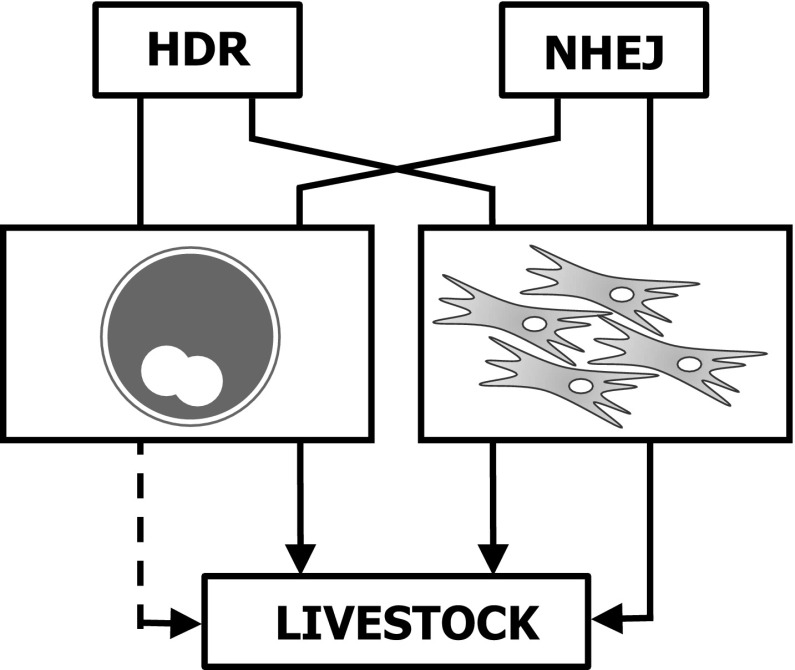
Routes to genome edited livestock. Designer nucleases have been successfully used to modify both zygotes and somatic cells. Modification and selection of fibroblasts coupled with SCNT has resulted in the generation of HDR and NHEJ edited livestock. NHEJ edited animals have been produced via zygote CPI whereas, to date, HDR edited animals have not been reported from edited zygotes

**Fig. 2:**
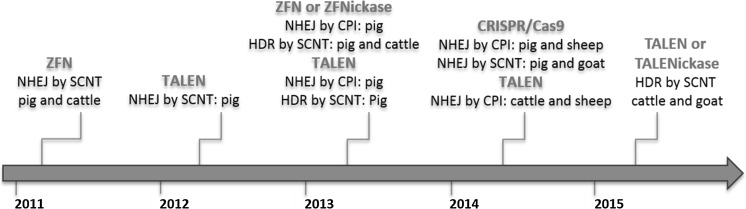
The utility of double strand breaks generated by genome editors. A cartoon depiction of the double strand break (DSB) repair mechanisms. Non homologous end joining (NHEJ) is an error prone process that re-joins the end of the DSB, often resulting in small insertions/deletions (*blue*) and subsequent gene disruption. Homology dependent repair is a faithful process that uses a homologous template to repair the DSB. Providing a repair template, either as a single stranded oligonucleotide or double stranded DNA, allows specific modifications (*green*) to be introduced to the genome. Creation of simultaneous DSBs flanking a region of the genome can result in deletion of the intervening sequence (*yellow*) and repair of the DSBs by either NHEJ or HDR. (Color figure online)

Following generation of a DSB at the desired locus, repair can occur in one of two ways; non-homologous end joining (NHEJ) or homology dependent repair (HDR; Fig. 1). In most cases of DSBs are repaired by NHEJ, with the two ends of the break being brought together and ligated. As a consequence of endogenous nuclease activity at the cut site this process is error prone and often results in the introduction small insertions/deletions (indels) at the repair site (Kanaar et al. 1998). Alternatively, if a repair template is provided in *trans*, evoking HDR in addition to NHEJ, the introduction of desired changes to the sequence at the targeted locus can be achieved (Kanaar et al. 1998). Deletion of regions of the genome can be achieved by generating of a pair of DSBs flanking the region to be deleted and their subsequent repair by NHEJ (Carlson et al. 2012; He et al. 2015b; Whitworth et al. 2014; Fig. 2).

Nickases are modified nucleases that only cut one strand of DNA, and mutagenesis of both FokI and Cas9 has resulted in nickase versions of these enzymes. By designing reagents such that staggered nicks are created at the target site (e.g. 2 pairs of ZFNickases), a DSB still occurs (Kim et al. 2012) and is repaired by either NHEJ or HDR. One potential advantage of this approach is that binding at off-target sites results in a nick rather than a DSB, with subsequent repair by the break excision repair (BER) pathway which leaves no mark on the genome. It is too early to know how useful the nickases strategy will be given the reduced efficiency associated with this Cas9 variant but the theoretical promise of reduced off-targets seems to be real (Ren et al. 2014; Frock et al. 2015).

## So much achieved in such a short period of time

**Fig. 3:**
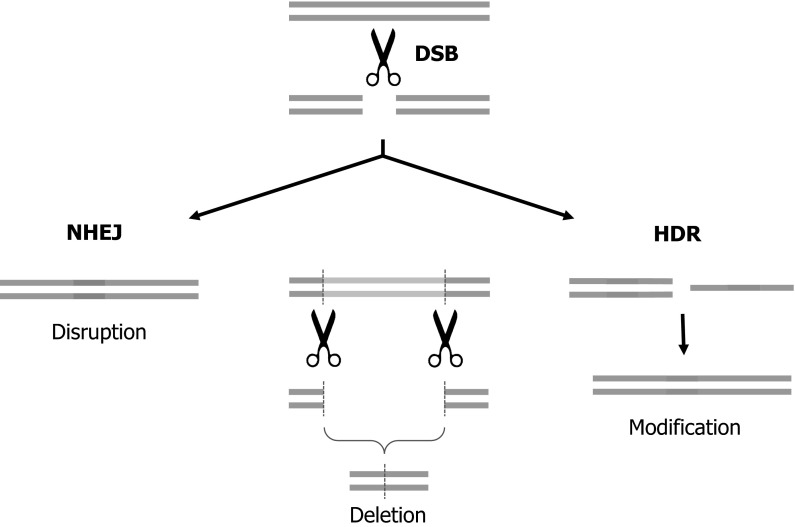
A Timeline of genome edited livestock over the past 5 years highlighting specific milestones

In the last 5 years, genome editors have been used to mediate the generation of more than 300 edited pigs, cattle, sheep and goats (Table 1). These animals can potentially serve as organ donors (Hauschild et al. 2011; Li et al. 2015), disease models (Tan et al. 2013), bioreactors (Liu et al. 2013) or founder animals of genetic lines with enhanced productivity (Proudfoot et al. 2015) or disease resistance traits (Lillico et al. 2013; Wu et al. 2015). To achieve this, our community has been progressively expanding the livestock genome engineering toolbox to include state-of-the-art technologies, first ZFNs (Yang et al. 2011; Whyte et al. 2011), then TALENs (Carlson et al. 2012) and CRISPR/Cas9 (Hai et al. 2014; Fig. 3).

**Table 1:** A list of published pigs, cattle, sheep and goats ever generated by genome editors

Gene(s)*	Editor	Route	Genotypes**	E.T./R/P***	Live/total born	F0 edited/live^†^	References
*NHEJ*							
Pig							
PPARγ	ZFN	SCNT	±	1340/8/4	10/10	2/10	Yang et al. (2011)
α 1,3GT	ZFN	SCNT	±, −/−	272/3/2	2/2	2/2	Hauschild et al. (2011)
eGFP	ZFN	SCNT	−/−	315/2/2	7/7	6/7	Whyte et al. (2011)
LDLR	TALEN	SCNT	−/−	n.a./9/7	18/22	18/18	Carlson et al. (2012)
α 1,3GT	ZFN	SCNT	±, −/−	304/3/2	3/4	3/3	Li et al. (2013)
RELA	ZFN	CPI	−/−	109/3/2	9/9	1/9	Lillico et al. (2013)
RELA	TALEN	CPI	±, ±/−, =/−	393/11/6	41/46	5/41	Lillico et al. (2013)
CMAH	ZFN	SCNT	±	431/2/2	11/13	11/11	Kwon et al. (2013)
IL2RG	ZFN	SCNT	−/Y	199/2/2	4/4^a^	4/4	Watanabe et al. (2013)
α 1,3GT CMAH	ZFN	SCNT	−, −/−, −	477/4/1	4/5	4/4	Lutz et al. (2013)
DAZL	TALEN	SCNT	−/−	n.a./3/2	3/5^c^	3/3	Tan et al. (2013)
α 1,3GT	TALEN	SCNT	−/−	1919/7/2	3/4	3/3	Xin et al. (2013)
α 1,3GT	ZFN	SCNT	−/−	2093/11/8	15/15	3/15	Bao et al. (2014)
RAG1	TALEN	SCNT	−/−	1285/9/6	12/24	9/12	Huang et al. (2014)
RAG2	TALEN	SCNT	±, −/−	3633/15/7	15/18	13/15	Huang et al. (2014)
RAG2	TALEN	SCNT	±, −/−	1903/9/9	22/31	13/13^d^	Lee et al. (2014)
GHR	TALEN	HMC	−/−	654^e^/6/n.a.	10/12	7/10	Li et al. (2014)
DJ-1	TALEN	SCNT	±, −/−	687/5/1	3/4	3/3	Yao et al. (2014)
vWF	CRISPR/Cas9	CPI	±/− , =/−	76/5/3	16/16	11/16	Hai et al. (2014)
SLA-1,2,3	CRISPR/Cas9	SCNT	−, −, −/− ,−, −, −	265/2/2^b^	3/3	3/3	Reyes et al. (2014)
CD163	CRISPR/Cas9	SCNT	−/−	2734/13/8	37/39	34/37	Whitworth et al. (2014)
CD1d	CRISPR/Cas9	SCNT	−/−	1055/5/4	13/13	12/13	Whitworth et al. (2014)
CD163	CRISPR/Cas9	CPI	−/−	96^e^/2/1	4/4	4/4	Whitworth et al. (2014)
CD1d	CRISPR/Cas9	CPI	−/−, =/−	110^e^/2/1	4/4	4/4	Whitworth et al. (2014)
TYR	CRISPR/Cas9	SCNT	−/−	1705/7/4	18/18	15/18	Zhou et al. (2014)
PARK2, PINK1	CRISPR/Cas9	SCNT	−, −/−, −	1729/10/4	18/20	18/18	Zhou et al. (2014)
IgM	CRISPR/Cas9	SCNT	−/−	500^e^/5/2	3/5	3/3	Chen et al. (2015)
PKD1	ZFN	SCNT	±	4987/13/5	20/25	13/20	He et al. (2015a)
α 1,3GT, CMAH iGb3S	CRISPR/Cas9	SCNT	−, −, −/−, −, −	179/2/2	10/12	5/10^f^	Li et al. (2015)
Npc1l1	CRISPR/Cas9	CPI	=/−	105/4/2	12/12	12/12	Wang et al. (2015)
Cattle							
BLG	ZFN	SCNT	−/−	995^e^/119/50	8/8	8/8	Yu et al. (2011)
GDF8	ZFN	SCNT	−/−	1336^e^/123/35	n.a./18	2/n.a.	Luo et al. (2014)
GDF8	TALEN	CPI	±/−	20^e^/11/2	2/4	½	Proudfoot et al. (2015)
Sheep							
GDF8	CRISPR/Cas9	CPI	±/−	213/55/31	35/35	2/35	Han et al. (2014)
GDF8	TALEN	CPI	±	26^e^/9/8	12/12	1/12	Proudfoot et al. (2015)
Goat							
GDF8	CRISPR/Cas9	SCNT	−/−	269/21/7	3/3	3/3	Ni et al. (2014)
*HDR*							
Pig							
CMAH	ZFN	SCNT	−/Neo	1619/7/4	7/7	5/7	Kwon et al. (2013)
DAZL	TALEN	SCNT	−/in4	n.a./3/2	2/3	2/2	Tan et al. (2013)
APC	TALEN	SCNT	in4/in4	n.a./3/2	5/6	5/5	Tan et al. (2013)
Cattle							
CSN2	ZFN or ZFNickase	SCNT	+/lst	1671^e^/559/140	14/19	14/14	Liu et al. (2013)
CSN2	ZFN	SCNT	+/hLYZ	236^e^/118/20	5/5	5/5	Liu et al. (2014)
MAT1A-SFTPA1 g	TALENickase	SCNT	+/SP110	465^e^/147/50	23/23	13/13	Wu et al. (2015)
Goat							
BLG	TALEN	SCNT	−/hLF^g^	n.a.	5/n.a.	2/5	Cui et al. (2015)

Publications were collected by searching the databases of Google Scholar and PubMed with keywords “ZFN” or “zinc finger nuclease”, “TALEN” or “TAL effector nuclease”, or “Cas9” in combination with “pig”, “cattle”, “sheep”, or “goat”. We hope that all published work by our dear colleagues are included as of early July 2015; we apologize if yours is unintentionally left out

*n.a.* not available, *SCNT* somatic cell nuclear transfer, *CPI* cytoplasmic injection, *HMC* hand-made cloning, *NHEJ* non-homologous end joining, *HDR* homology directed repair

* *APC* Adenomatous polyposis coli, *α 1,3GT* α1,3-galactosyltransferase (GGTA1), *BLG* beta-lactoglobulin, *CD163* cluster of differentiation 163, *CD1d* cluster of differentiation 1d, *CMAH* CMP-*N*-acetylneuraminic acid hydroxylase, *CSN2* b-casein, *DAZL* deleted in azoospermia-Like gene, *DJ*-*1* protein deglycase DJ-1 or Parkinson disease protein 7, *GDF8* growth differentiation factor 8 or Myostatin, *GHR* growth hormone receptor, *hLYZ* human lysozyme, *iGb3S* iGb3 synthase, *IgM* immunoglobulin M, *PKD1* polycystin-1, *IL2RG* interleukin-2 receptor gamma, *LDLR* low density lipoprotein receptor, *lst* lysostaphin, *MAT1A*-*SFTPA1* *g* introgenic sequence between gene MAT1A and SFTPA1 g, *Npc1l1* Niemann-Pick C1-Like 1, *PINK1* PTEN-induced putative kinase 1, *PPARγ* peroxisome proliferator-activated receptorgamma, *RAG1/2* recombination activation gene ½, *RELA* p65, *SLA*-*1,2,3* swine leukocyte Ags 1,2, and 3, *TYR* tyrosinase, *PARK2* gene encoding parkin, *vWF* von Willebrand factor

** ± One allele modified by NHEJ, −/− both alleles modified by NHEJ, =/− mosaicism with up to 5 genotypes but no wt sequence in a single animal, −/Y X-chromosome gene targeted in male cells, ±/− mosaicism with up to 6 genotypes including wt sequence; −/Neo, −/in4, −/hLF: one allele modified by NHEJ while the other knockout by a Neo cassette, a 4 bp insertion or a human lactoferrin expression cassette; +/lst, +/hLYZ, +/SP110: mono-allelic insertion of a transgene, lysostaphin, human lysozyme, or SP110 nuclear

body protein gene

*** E.T./R/P: total embryos transferred/total recipients/total pregnancies

^†^ Only animals generated by the initial cloning rather than re-cloning are listed

^a^ These are full term foetuses delivered by C-section

^b^ This is accompanied by re-cloning using fibroblasts isolated from an aborted pregnancy

^c^ The donor cells with NHEJ events were mixed with those with HDR alleles for cloning

^d^ Genotyping of the rest of live born piglets were not described

^e^ Only blastocysts were transferred

^f^ The rest of the animals have NHEJ events at least in 2 out of 6 alleles

^g^ −/hLF animals were generated on the **±** cells background

**Fig. 4:**
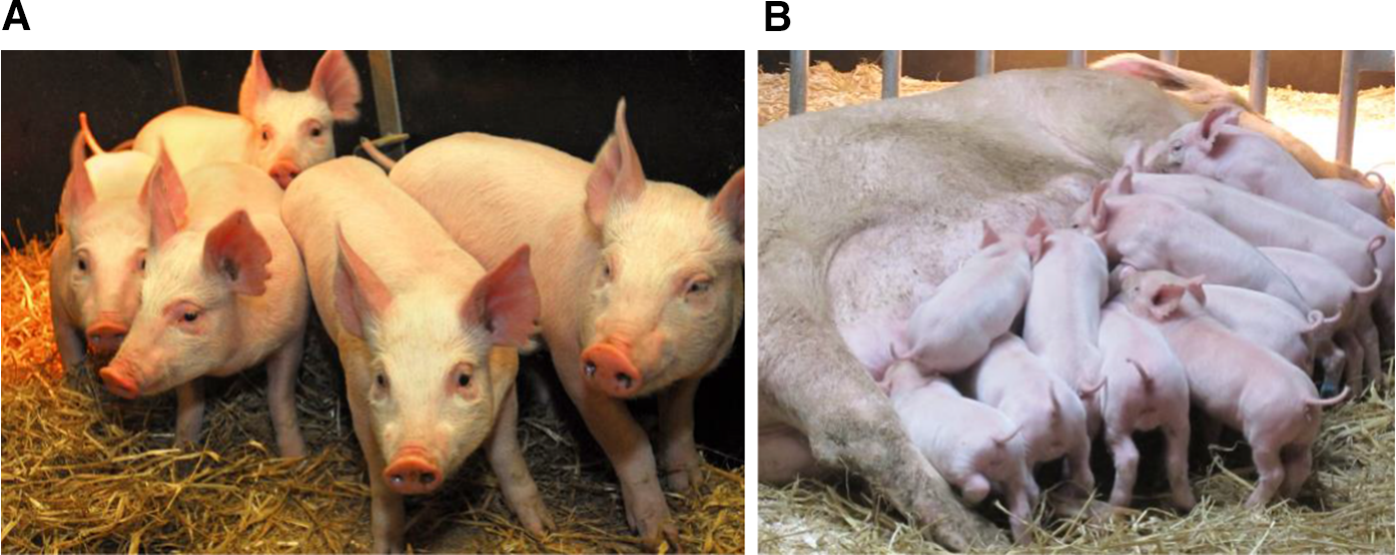
Live genome edited pigs produced by TALEN injection into zygotes. **a** Founder NHEJ animals born 2012 (Lillico et al. 2013). **b** Third generation piglets derived from NHEJ founder animals

The creation of the first genome edited animals relied on the modification of primary cells which were then used as nuclear donors for embryo reconstruction in SCNT (Hauschild et al. 2011; Carlson et al. 2012; Fig. 3). An efficient alternative, direct modification of zygotes by cytoplasmic injection (CPI) of the editors, soon followed (Fig. 4a; Lillico et al. 2013; Hai et al. 2014) rekindling the microinjection skills used for the first transgenic livestock (Fig. 3). Adding to these initial reports that described NHEJ events, the ability to introduce defined sequences into a targeted locus through HDR, using either single strand DNA oligonucleotides (ssODN; Tan et al. 2013) or plasmids as repair templates (Liu et al. 2013; Wu et al. 2015) has been demonstrated for livestock. Rather than depending on random changes at the target site introduced by the error prone NHEJ repair pathway, these defined sequence changes allow either more precise gene knockout or targeted integration of various transgenes, and importantly make allele swapping possible (Fig. 2).

SCNT has been the primary method to deliver nuclease mediated genetic changes into livestock. To date, 33 out of 43 reported successes utilise SCNT and resulted in 267 edited live animals (Table 1). This focus on SCNT reflects the lead position this technology has had for the last couple of decades in livestock biology, especially for pigs, since one can apply nucleases then pre-select editing events in vitro. At time of writing, SCNT is the only published way to create livestock with defined changes by HDR (Fig. 3).

The combination of genome editors and SCNT has proven to be powerful. It is possible to obtain cells with bi-allelic modifications in one-step by marker-free dilution cloning (Tan et al. 2013), or if necessary FACS sorting (Whyte et al. 2011; Reyes et al. 2014) or drug selection (Liu et al. 2013; Wu et al. 2015). Moreover, simultaneous targeting of different genes has allowed bi-allelic modification of up to three genes at the same time (Reyes et al. 2014; Li et al. 2015). Cloning using such cells has resulted in an average 76 % editing rate in live born pigs (Table 2); some of these animals contain gene inactivating indels resulting from NHEJ (Lutz et al. 2013; Whitworth et al. 2014) or ssODN mediated HDR (Tan et al. 2013), while others have site-specific insertions of transgenes by HR (Kwon et al. 2013; Wu et al. 2015; Cui et al. 2015).

**Table 2:** A summary of edited animals created by SCNT or CPI

	Edited/live born	Pregnancy rate
*A. Success percentages*		
Pig		
SCNT	76 % (179/237)	55.3 % (62/112)
CPI	37 % (29/78)	56.5 % (13/23)
Cattle, sheep and goats		
SCNT	81 % (43/53)	27.7 % (267/964)
CPI	8.2 % (4/49)	54.7 % (41/75)

	Embryos/recipient	Embryos/edited live	Live born/pregnancy	Edited/pregnancy
*B. Ratio of desired outcome across different stages of methodology*				
Pig				
SCNT	207 (23,216/112)	130 (23,216/179)	3.8 (237/62)	2.9 (179/62)
CPI	30 (688/23)	24 (688/29)	6 (78/13)	2.2 (29/13)
Cattle, sheep and goats				
SCNT	NA	244 (10,510/43)	0.20 (53/267)	0.16 (43/267)
CPI	NA	128 (513/4)	1.2 (49/41)	0.10 (4/41)

Only entries with complete records in Table 1 are used for analysis. Pig SCNT data were calculated from 21 entries whereas CPI data were added up from Lillico et al. (2013), Hai et al. (2014) and Wang et al. (2015). Pig CPI data from Whitworth et al. 2014 were not included in the analysis because injected embryos were cultured to blastocysts before transfer. For other livestock species, SCNT data were calculated from five reports whereas CPI data were generated from Han et al. (2014) and Proudfoot et al. (2015)

*NA* not applicable because in some experiments embryos were transferred shortly after reconstruction or injection while in others embryos were cultured to blastocysts

Production of editor modified animals via SCNT is hugely successful, but remains tied to the drawbacks associated with cloning. In the published reports using editors and SCNT, cloning efficiency has been low, being only 1.2 % (278/23,216) for pigs and 0.6 % (58/10,510) for other livestock (Table 2). On average, production of one edited live pig requires reconstruction of 130 embryos, which would be challenging without ready access to abattoir-sourced oocytes. The cloning efficiency is partly affected by donor cell quality: prolonged culture and multiple manipulations of the cells decrease their efficiency as nuclear donors. Because of this, some studies have required re-cloning to obtain more founder animals (Hauschild et al. 2011), especially when several genes were targeted simultaneously (Lutz et al. 2013; Reyes et al. 2014). In addition, SCNT (even in the absence of nuclease treatment) is often associated with problems such as birth defects, abortions and early postnatal death (Keefer 2015).

To circumvent issues associated with cloning, some research groups have adopted direct microinjection of editing reagents to the cytoplasm of zygotes. This approach has been effective in generating edited livestock animals using all three designer nucleases systems in pigs (Lillico et al. 2013; Hai et al. 2014; Whitworth et al. 2014; Wang et al. 2015), cattle (Proudfoot et al. 2015) and sheep (Han et al. 2014; Proudfoot et al. 2015). Although application of CPI has not been as widely used as SCNT in livestock genome editing (41 edited animals by CPI vs 267 animals by SCNT (Table 1), its use is gaining momentum. This shift presumably reflects the simplicity and versatility of CPI over SCNT. While the editing efficiency in live-born animals is lower for CPI (in pigs 37 % for CPI vs 76 % for SCNT), reflecting the lack of selection that takes place during the in vitro phase of SCNT, CPI only requires an average of 24 embryos to produce one edited pig, a five-fold improvement on that currently reported for SCNT (Table 2). Furthermore, one of the biggest advantages of CPI is that it can be applied to zygotes from any desired parental cross, maintaining genetic diversity in progeny. By contrast, SCNT tends to use genetic material from a clonal cell population, resulting in offspring that are genetically identical to the donor cells and thus requiring subsequent outcrossing to maintain genetic variation. SCNT, however, enables selection of a specific mutation prior to production of animals and potentially enables access to genetic lines where the correct embryo donors are not readily available. Thus, both CPI and SCNT offer the opportunity to utilise the range of editing events that are produced at a given locus.

Given that there is scope for improving the proportion of live-born edited animals following CPI, we anticipate that the numbers of both donor and recipient animals required per edited offspring will continue to be reduced. Combined with freedom from cloning related problems and greater choice in genetic background, CPI may prove to be the more compelling method—at least for agricultural applications. In the meantime there are two areas that require further investigation; founder mosaicism and efficient HDR. Mosaicism is commonly observed in edited animals produced by CPI (Lillico et al. 2013; Han et al. 2014; Proudfoot et al. 2015), and while this could be problematic with respect to analysis of phenotype in the founder generation, our group routinely breed CPI generated F0 pigs (homozygous, heterozygous or mosaic) to produce F1 offspring with the desired genotype (Fig. 4b; unpublished data). Other groups have confirmed germline transmission from mosaics by germ cell genotyping (Hai et al. 2014; Wang et al. 2015). Ideally mosaicism following CPI needs to be reduced; options currently being explored include maximizing editor concentration while controlling toxicity and off-target mutations or alternatively delivering Cas9 as protein rather than mRNA. HDR mediated editing via CPI has yet to be reported in live-born animals. However, given the high efficiency of multiplexing editing events in rodents (Wang et al. 2013; Yang et al. 2013), the recent success by co-injecting editors and ssODNs into in vitro cultured bovine embryos (Wei et al. 2015), and data from our own lab indicating that allele swap animals can be produced in zygotes (unpublished results), this is unlikely to prove a significant limitation.

## Expanding current trends

Although most of the initial goals in livestock genetic engineering focused on agriculture (Pursel et al. 1989), the combination of rudimentary (although at the time innovative) tools, insufficient knowledge of the genetic consequence of modifying the initial targets (e.g. growth hormone: Pursel et al. 1989) and lack of public support lead to a dearth of funding, both public and commercial, in this area. The result was the development of applications on the biomedical-biotechnological interface: animal bioreactors, xenotransplantation and, more recently, large animal models of human disease (Jänne and Alhonen 1998; Kind and Schnieke 2008; Cooper et al. 2014; Kostic et al. 2013). Given this spectrum of applications, impacts of genome editing may be quite variable. For animal bioreactor projects genome editing technology can enable refined expression strategies and possibly be used to augment post-translational effects. While editing technology could accelerate the development of even more animal resources for xenotransplantation, it is perhaps at the interface of these two applications that genome editing technology may come to the fore. The remarkable report of chimeric mice that carried a rat cell derived pancreas (Kobayashi et al. 2010) reignited enthusiasm for the possibility of animals producing human organs, or at least human cells. The passage of time since the original report may have dampened some of the initial optimism, but effort in this direction continues. Success would be spectacular.

Within the biomedical arena there is a growing realisation that small animal models, although delivering great mechanistic insight into disease in the model organism, are insufficient as translational tools for converting this knowledge from bench research to bedside applications. In many situations we would benefit from the use of larger animals to model both the development of disease pathology and the testing of intervention strategies. Genome editing enables the mutation of endogenous livestock gene homologues of known causative or associated human disease loci. Although there are no projects yet that have fully exploited this new technology, there are already a growing number of projects based on the ‘older’ transgenic technologies that have demonstrated that this belief in large animal disease models is justified (Wolf et al. 2014; Aigner et al. 2010).

## A path to agriculture

Man has pursued the selective breeding of animals for a long time. Initially our Mesolithic ancestors identified animals for their ability to breed in captivity based on aspects of temperament and social structure. Now engineering enables the targeting introduction of mutations providing increased genetic variation for the animal breeder to utilise. Although some agricultural applications of GM have been pursued by the research community, industry has been shy of engaging with the traditional transgenic technologies. That reluctance to directly engage with projects involving livestock appears to have eased with the advent of genome editing technology.

Historically animal breeding relied on selection by individual farmers of breeding stock with visibly desirable traits. Bioinformatic use of genetics allows a more refined selection process but still relies on selection of randomly segregating loci that are predicted to underlie advantageous traits (Daetwyler et al. 2013; Van Eenennaam et al. 2014; Hill 2014). Genome editing enables introgression of single genetic loci in contrast to current breeding regimes. It enables access to inaccessible variation: variation that doesn’t exist in a given breeding population (Lillico et al. 2013), i.e. variation out-with the breeding gene pool (a currently discussed scenario is introgression of the polled trait into elite Holstein cattle). It also offers solutions to non-segregation of beneficial and undesirable traits due to physical proximity of the underlying loci in the genome. Alternatively, genome editing offers a route to eliminate deleterious alleles from a gene pool.

Genome editing could also be used to modify quantitative traits that are affected by many loci (associated with a number of quantitative trait nucleotides). In the natural state low levels of recombination will limit the rate favourable alleles will arise together in selected individuals. This presents challenges for breeding regimes aiming to improve such a quantitative trait. Simulations show that even relatively modest multiplexing of genome editing targets has great potential for increasing the response to selection in breeding programmes over improvement by genomic selection alone (Jenko et al. 2015).

Many sectors of the animal agriculture industry, including those working with pigs, cattle, and chickens are now actively engaged with the technology through collaborations with the academic community. There remain the perceived barriers to adoption of these technologies in animal agriculture including public opinion and the regulatory environment; but these factors are in flux. Public opinion is often largely influenced by a vocal minority of concerned members of our society, but within society there also exists support and interest in the potential for genetically engineered livestock to make a contribution to the global challenge of food security. This is increasingly being reflected by the constructive media portrayal of this biotechnology. In turn, the political mood is also changing; for example, the headline in the UK press early 2015 “EU regulation on GMOs not ‘fit for purpose’ say UK MPs”. Nevertheless, at time of writing, we still see the regulatory approval for arguably the world’s lead GM product (the AquAdvantage salmon) in the quagmire that occurs when politics overrides the scientific evidence-based position and the approved regulatory process (Van Eenennaam and Muir 2011).

It is hoped that genome edited livestock (Fahrenkrug et al. 2010), with their lack of introduced transgenes, will find a smoother path through the regulatory system. In plants the regulatory bodies view this enzyme-based technology as they would chemical or radiation induced mutagenesis; the latter two methods have a long history of unregulated use but lack the specificity afforded by genome editing tools. We believe that in this regulatory environments, and based on the specificity and ‘footprintless’ nature of genome editing, gene editing animals will successfully navigate both the political and regulatory landscape.

## What is next for genome engineering

Transgenic livestock were first produced in the mid-1980s and subsequently through the use of HR and SCNT, gene targeting in large animals has been possible since the late 1990s (Clark and Whitelaw 2003). Gene editing brings nothing conceptually new to the table. Rather, this set of tools greatly facilitates what was already possible with traditional methods, increasing the rate at which new projects can be delivered. This is fast becoming reality with an incredible pace of progress being evident—for those engaged with the literature there is the real feeling that as many new lines of engineered large animals have been produced in the last few years as in the previous three decades.

Some believe genome editing tools provide the best imaginable technology for mutating the germline. Indeed it is hard at the moment to imagine what could be better. Nevertheless there are remaining challenges. We need to improve efficiency of editing within a given population of cells (destined for SCNT) and in the zygote and overcome mosaicism. In our work with zygotes we regularly achieve 30 % editing frequency with delivery of editors—ZFN, TALEN and CRISPR/Cas9—to the cytoplasm of livestock. We should aspire to at least >50 % and why not frequencies approaching or even achieving 100 %.

We need to further refine our predictive editor design algorithms. The scope to expand the repertoire of editing reagents continues through the development of Cas9 variants (Kleinstiver et al. 2015; Ran et al. 2015) and meganucleases (Ménoret et al. 2013) is already materializing with the promise that many more will be forthcoming. For real utility in addressing multi-quantitative nucleotides underlying a quantitative locus we will need the ability to multiplex editing events. Conceptually this could be challenging, given the possibility for multiple target sites in a given genome to undergo inter-site events, resulting in deletion or other forms of recombination, yet the production mice with of three consecutively edited sites has been reported.

For reasons both practical and public perception based, the concern about off-targets must be addressed. Off-target effects occur because the editing complex relies on base-recognition affinity for targeting but can cut at a lower frequency at similar non-target sites (analogous to the star activity exhibited by restriction endonucleases). Although a much discussed point, the emerging evidence now suggest that off-targets may be rare events in mice (Iyer et al. 2015), supporting previous human cell data (Kim et al. 2015). Nevertheless there is probably still room for improvement, although the debate about what could be tolerated for a given application remains to be resolved. To address this aspect a multitude of strategies are being evaluated. For example, masking Cas9 with a fusion peptide preventing activity until cleaved by a small molecule (Davis et al. 2015), expanding the TALEN RDV repertoire (Miller et al. 2015), dimerisation of the editing enzyme (Wright et al. 2015; Zetsche et al. 2015), use of nickases which cause single strand-breaks rather than double-strand breaks therefore evoking different DNA repair processes (Frock et al. 2015) and, further in this vein, the inhibition of NHEJ (Maruyama et al. 2015; Chu et al. 2015).

Continued development of genome editing tools will accelerate livestock biotechnology through their ease of use. Where it took several painfully taxing years for several groups around the world to produce alpha-1,3 galactosyltransferase null pigs, this can now rapidly and easily be achieved through the use of genome editors. This ‘catch-up’ phenomenon is not unique to these tools and reflects all aspects of technological advance (most obvious in our ability to sequence genomes). And like other significant technology leaps, this results is more and greater diversity in applications. Concerns of off-targets are reducing and the political landscape increasingly supportive. Although currently there is a crowded intellectual property landscape enveloping the genome editors (e.g. Sherkow 2015), paths through this legal environment will resolve with time. We are only at the start of this wave of advance—the world for livestock biotechnology is about to get very exciting.
